# Report from the 4th Cardiovascular Outcome Trial (CVOT) Summit of the Diabetes & Cardiovascular Disease (D&CVD) EASD Study Group

**DOI:** 10.1186/s12933-019-0822-4

**Published:** 2019-03-11

**Authors:** Oliver Schnell, Eberhard Standl, Doina Catrinoiu, Baruch Itzhak, Nebojsa Lalic, Dario Rahelic, Jan Skrha, Paul Valensi, Antonio Ceriello

**Affiliations:** 1 Forschergruppe Diabetes e. V., Ingolstaedter Landstraße 1, Neuherberg, 85764 Munich, Germany; 2 Internal Medicine Department, Clinical County Emergency Hospital Constanta, Tomis Blvd. No. 145, 900591 Constanța, Romania; 3 Clalit Health Services and Technion Faculty of Medicine, 3 Zivoni Street, Haifa, Israel; 4 Clinic for Endocrinology, Diabetes and Metabolic Diseases, Clinical Center of Serbia, Faculty of Medicine, University of Belgrade, Dr Subotica 13, Belgrade, 11000 Serbia; 5 Diabetes and Metabolic Disorders, Dubrava University Hospital, Avenija Gojka Šuška 6, 10000 Zagreb, Croatia; 6 3rd Department of Internal Medicine, 1st Faculty of Medicine, Charles University, U Nemocnice 1, 128 08 Prague 2, Czech Republic; 7 Department of Endocrinology Diabetology Nutrition, CINFO, CRNH-IdF, Jean VERDIER Hospital, Paris 13 University, Avenue du 14 Juillet, 93140 Bondy, France; 8 Institut d’Investigacions Biomèdiques August Pi i Sunyer (IDIBAPS) and Centro de Investigación Biomedica en Red de Diabetes y Enfermedades Metabólicas Asociadas (CIBERDEM), Barcelona, Spain; 9 Department of Cardiovascular and Metabolic Diseases, IRCCS MultiMedica, Via Milanese 300, 20099 Sesto San Giovanni, MI Italy

**Keywords:** Cardiovascular risk, Diabetes, CVOT, CARMELINA, DECLARE-TIMI 58, Harmony Outcomes, ODYSSEY OUTCOMES

## Abstract

The 4th Cardiovascular Outcome Trial (CVOT) Summit of the Diabetes & Cardiovascular Disease (D&CVD) EASD Study Group was held in Munich on 25–26 October 2018. As in previous years, this summit served as a reference meeting for in-depth discussions on the topic of recently completed and presented CVOTs. This year, focus was placed on the CVOTs CARMELINA, DECLARE-TIMI 58 and Harmony Outcomes. Trial implications for diabetes management and the impact of the new ADA/EASD consensus statement treatment algorithm were highlighted for diabetologists, cardiologists, endocrinologists, nephrologists and general practitioners. Discussions evolved from CVOTs to additional therapy options for heart failure (ARNI), knowledge gained for adjunct therapy of type 1 diabetes and, on the occasion of the 10 year anniversary of the FDA’s “Guidance for Industry: “should CVOTs be continued and/or modified?” The 5th Cardiovascular Outcome Trial Summit will be held in Munich on 24–25 October 2019 (http://www.cvot.org).

## Background

Coronary heart disease, cerebrovascular disease and peripheral arterial disease of atherosclerotic origin, collectively termed atherosclerotic cardiovascular disease (ASCVD), are the major cause of mortality in patients with diabetes mellitus [1, 2]. Diabetes patients experience an up to 50% increased risk of cardiovascular (CV)-related death [3]. A variety of studies has shown that an improvement in glycaemic control can positively impact long-term CV disease (CVD) risk in patients with type 2 diabetes mellitus (T2DM) [4, 5]. However, other trials like the UGDP [6] and ACCORD trial [7], as well as studies on muraglitazar [8] and rosiglitazone [9], raised concerns for elevated CV risk [10]. This prompted the Food and Drug Administration (FDA) to release a “Guidance for Industry” in 2008, for the evaluation of CV safety of new antidiabetic therapies in T2DM in order to prevent an inacceptable increase of CV risk [11]. In consequence, CV outcome trials (CVOTs) for glucose lowering therapies were introduced. In CVOTs, combined CV endpoints are evaluated as primary outcome, usually including CV mortality, non-fatal myocardial infarction (MI) and non-fatal stroke (3-point major adverse CV event, 3P-MACE). Some trials include the hospitalisation rate for unstable angina pectoris as additional primary outcome (4P-MACE). Secondary outcomes often include hospitalisation for heart failure (HF), death from CV causes, all-cause mortality and renal outcomes. Since 2008, every newly approved glucose lowering drug has undergone a CVOT to evaluate its CV safety (hazard ratio (HR) < 1.8) [12]. So far, this has encompassed four main classes of substances: (1) dipeptidyl-peptidase-4 inhibitors (DPP-4i) (SAVOR-TIMI 53—saxagliptin; EXAMINE—alogliptin; TECOS—sitagliptin); (2) glucagon-like peptide-1 receptor agonists (GLP-1 RA) (ELIXA—lixisenatide; LEADER—liraglutide; SUSTAIN-6—semaglutide; EXSCEL—exenatide); and (3) sodium/glucose co-transporter-2 inhibitors (SGLT-2i) (EMPA-REG OUTCOME—empagliflozin; CANVAS—canagliflozin) as well as two insulins (ORIGIN—insulin glargine; DEVOTE—insulin degludec) [13–23], previously summarised by Schnell et al. [24, 25].

In 2018, the list of published CVOTs was further increased with CARMELINA (linagliptin, DPP-4i) [26], Harmony Outcomes (albiglutide, GLP-1 RA) [27] and DECLARE-TIMI 58 (dapagliflozin, SLGT-2i) [28]. In addition, a CV safety study for alirocumab (ODYSSEY OUTCOMES), a proprotein convertase subtilisin/kexin type-9 inhibitor (PCSK-9i), was published [29]. As in previous years [30–32], we present and summarise the key aspects discussed at the 4th CVOT Summit in October 2018.

## Updates on CVOTs

A summary of characteristics and results of CVOTs published in 2018 is listed in Tables 1 and 2.

**Table 1:** Overview of basic characteristics of CVOTs studies completed in 2018

Study name	Study status	Drug	Drug class	Intervention	Primary outcome	n	Follow up [years]	Start and end date	Clinicaltrials. gov ID
CARMELINA	Completed	Linagliptin	DPP-4 inhibitor	Linagliptin 5 mg daily vs. placebo	CV death, non-fatal MI, non-fatal stroke	6.980	4.5	07.2013–01.2018	NCT01897532
Harmony Outcomes	Completed	Albiglutide	GLP-1 receptor agonist	Albiglutide 30 mg to 50 mg weekly vs. placebo	CV death, non-fatal MI, non-fatal stroke	9.574	≥ 1.5	07.2015–02.2018	NCT02465515
DECLARE-TIMI 58	Completed	Dapagliflozin	SGLT-2 inhibitor	Dapagliflozin 10 mg daily vs. placebo	CV death, MI, ischemic stroke, hospitalisation due to heart failure	17.276	6	04.2013–07.2018	NCT01730534
ODYSSEY OUTCOMES	Completed	Alirocumab	PCSK9 inhibitor	Alirocumab 75 mg or 150 mg two-weekly vs. placebo	CHD death, non-fatal MI, fatal and non-fatal ischemic stroke, unstable angina requiring hospitalisation	18.924	2.8	10.2012–01.2018	NCT01663402

**Table 2:** CVOTs completed in 2018: comparison of results vs. placebo

Cardiovascular endpoints	CARMELINA		Harmony Outcomes		DECLARE-TIMI 58		ODYSSEY OUTCOMES	
	Class	HR (95% CI) p-value	Class	HR (95% CI) p-value	Class	HR (95% CI) p-value	Class	HR (95% CI) p-value
Primary composite MACE	CV death, non-fatal MI or non-fatal stroke	1.02 (0.89–1.17) p < 0.001^a^ p = 0.74^b^	CV death, non-fatal MI or non-fatal stroke	0.78 (0.68–0.90) p < 0.0001^a^ p = 0.0006^b^	CV death, non-fatal MI or non-fatal stroke	0.93 (0.84–1.03) p = 0.17^b^	CV death, non-fatal MI, non-/fatal stroke or unstable angina	0.85 (0.78–0.93) p < 0.001
Cardiovascular death	Exploratory outcome	0.96 (0.81–1.14) p = 0.63	Secondary endpoint	0.93 (0.73–1.19) p = 0.578	Primary endpoint	0.83 (0.73–0.95)^c^ p = 0.005^c^	Secondary endpoint	0.88 (0.74–1.05) –
Myocardial infarction	Exploratory outcome	1.12 (0.90–1.14) p = 0.30	Secondary endpoint	0.75 (0.61–0.90) p = 0.003	Primary endpoint	0.89 (0.77–1.01) –	Additional endpoint	0.86 (0.77–0.96) –
Stroke	Exploratory outcome	0.88 (0.63–1.23) p = 0.45	Secondary endpoint	0.86 (0.66–1.14) p = 0.300	Primary endpoint	1.01 (0.84–1.21) –	Additional endpoint	0.73 (0.57–0.93) –
Hospitalisation for unstable angina	Exploratory outcome	0.87 (0.57–1.31) p = 0.50	–	– –	–	– –	Additional endpoint	0.61 (0.41–0.92) –
Hospitalisation for heart failure	Exploratory outcome	0.90 (0.74–1.08) p = 0.26	–	– –	Primary endpoint	0.73 (0.61–0.88) –	Additional endpoint	0.98 (0.79–1.20) –

	Event rate (%) active group		Event rate (%) active group		Event rate (%) active group		Event rate (%) active group	
Primary composite MACE	12.4		4.57		8.8		9.5	

	No. (%) p-value		No. (%) p-value		No. (%) p-value		No. (%) p-value	
Renal event	6.6^d^ 0.87		6 –		0.76^e^ –		– –	
Acute pancreatitis	0.3 –		< 1 –		– –		– –	
Severe hypoglycaemic events	3.0 –		1 –		0.7 0.02		– –	

^a^p-value for non-inferiority

^b^p-value for superiority

^c^Cardiovascular death or hospitalisation for heart failure

^d^Sustained ESRD, death due to kidney failure, or sustained decrease of ≥ 50% in eGFR from baseline

^e^Composite renal outcome

### DPP-4 inhibitors

The CARMELINA trial [26] investigated the effect of once-daily linagliptin on CV and kidney outcomes in patients with T2DM at high risk of CV and kidney events. With respect to kidney outcomes, CARMELINA was the first DPP-4i CVOT to investigate a composite kidney outcome in a statistically adequately powered manner [26]. Inclusion criteria for the 6979 patients comprised high risk of vascular events (e.g. history of MI, stroke or coronary artery disease) or impaired renal function with or without CV comorbidities [33]. In the primary endpoint (3P-MACE: CV mortality, non-fatal MI and non-fatal stroke), linagliptin showed CV safety (HR 1.02 (95% CI 0.89–1.17), p < 0.001 for non-inferiority) compared to placebo but did not demonstrate a CV benefit. No significant benefit was observed in the secondary kidney composite outcome (HR 1.04 (95% CI 0.89–1.22), p = 0.62) compared to placebo. Exploratory kidney and microvascular outcomes showed a significant reduction of albuminuria progression (HR 0.86 (95% CI 0.78–0.95), p = 0.003) and a significant reduction in the composite microvascular endpoint (HR 0.86 (0.78–0.95), p = 0.003) in the linagliptin group compared to placebo [26].

### GLP-1 receptor agonists

In the Harmony Outcomes CVOT, CV effects of once-weekly albiglutide in patients with T2DM were evaluated [27]. A total of 6493 participants with approximately 100% prior CVD was followed for a median of 1.6 years and assessed for 3P-MACE. With respect to the primary outcome (3P-MACE: CV mortality, non-fatal MI and non-fatal stroke), albiglutide showed superiority compared to placebo (HR 0.78 (95% CI 0.68–0.90), p = 0.0006; p < 0.0001 for non-inferiority). Statistically significant secondary outcomes included a reduced expanded composite outcome (death from CVD, non-fatal MI, non-fatal stroke or urgent revascularisation for unstable angina; HR 0.78 (95% CI 0.69–0.90), p = 0.0005) and a reduction of fatal or non-fatal MI (HR 0.75 (95% CI 0.61–0.90), p = 0.003). Incidences of acute pancreatitis, pancreatic cancer and medullary thyroid carcinoma did not differ between the albiglutide and placebo group [27].

### SGLT-2 inhibitors

CV safety of dapagliflozin was investigated in the DECLARE-TIMI 58 trial [28]. The trial encompassed 17,160 patients who were followed during a median of 4.2 years. A hitherto unique aspect of the DECLARE-TIMI 58 trial was its high proportion of patients in primary prevention, as 59.4% of the enrolled patients had no prior ASCVD. Dapagliflozin showed non-inferiority to placebo with respect to 3P-MACE (p < 0.0001 for non-inferiority), yet not superiority (p = 0.17 for superiority). As pre-defined co-primary superiority endpoint, a significant reduction of CVD death or hospitalisation for HF (HR 0.83 (95% CI 0.73–0.95), p = 0.005) was demonstrated. In addition, reduction of the renal composite endpoint (≥ 40% decrease in estimated glomerular filtration rate (eGFR) to < 60 mL/min/1.73 m^2^, new end-stage renal disease or death from renal or cardiovascular causes; HR 0.76 (95% CI 0.67–0.87)) and reduction of death from any cause (HR 0.93 (95% CI 0.82–1.04)) were observed. Adverse events included a significant increase of diabetic ketoacidosis (0.3% vs. 0.1%, p = 0.02), a significant increase in the rate of genital infections (0.9% vs. 0.1%, p < 0.001) but no increase in the risk of amputation with dapagliflozin compared to placebo [28].

### PCSK-9 inhibition

The ODYSSEY OUTCOMES study was designed to assess CV outcomes of alirocumab in 18,924 patients with prior acute coronary syndrome and low-density lipoprotein (LDL) cholesterol levels of at least 70 mg/dL, non-high-density lipoprotein (HDL) cholesterol levels of at least 100 mg/dL or an apolipoprotein B level of at least 80 mg/dL and who were receiving statin therapy at high or maximum tolerated dose [29]. 28.5% of the alirocumab study population had diabetes mellitus. Alirocumab significantly decreased CV outcomes (composite of death from coronary heart disease, non-fatal MI, fatal or non-fatal ischemic stroke, or unstable angina requiring hospitalisation; HR 0.85 (95% CI 0.78–0.93), p < 0.001) compared to placebo. Secondary endpoints included a significant reduction of any coronary heart disease event (death from coronary heart disease, non-fatal MI, unstable angina requiring hospitalisation, and an ischemia-driven coronary revascularisation procedure, HR 0.88 (95% CI 0.81–0.95), p = 0.001) and a significant reduction of 3P-MACE (HR 0.86 (95% CI 0.79–0.93), p < 0.001) [29]. The previously published study FOURIER investigated the CV safety of evolocumab in a set of 27,564 patients with ASCVD and LDL-cholesterol levels of 70 mg/dL or higher, who already were receiving statin therapy [34]. 5% of this study population were patients with diabetes. Evolocumab demonstrated a CV benefit compared to placebo, with a significant decrease in the primary endpoint (cardiovascular death, MI, stroke, hospitalization for unstable angina, or coronary revascularization; HR 0.85 (95% CI 0.79 – 0.92), p<0.001) and the key secondary endpoint (cardiovascular death, MI, or stroke; HR 0.80 (95% CI 0.73 – 0.88), p<0.001). Positive effects were particularly observed regarding the CV risk reduction in patients with T2DM [34].

### Angiotensin-receptor–neprilysin-inhibitors (ARNI)

HF has been shown to strongly correlate with diabetes with a significantly increased initial presentation.  Also, a dramatic increase of HF incidence was demonstrated corresponding to increasing age of diabetes patients, compared to individuals without diabetes [35]. At this summit, an additional option for the therapy of HF, the angiotensin-receptor–neprilysin-inhibition (ARNI) therapy, was discussed.

In the PARADIGM-HF trial, ARNI (valsartan/sacubitril) was compared to enalapril. A significant reduction in the primary composite endpoint (death from CV causes or first hospitalisation for worsening HF, death from CV causes, hospitalisation for HF and death from any cause) was observed [36]. A post hoc analysis of the PARADIGM-HF trial revealed that valsartan/sacubitril might have beneficial effects on the glucose metabolism in patients with known diabetes (98% T2DM) or an HbA1c ≥ 6.5% [37]. The recently presented PIONEER-HF trial revealed that valsartan/sacubitril, compared to enalapril, also has a significantly greater effect on the reduction of brain natriuretic peptide (NT-proBNP) among patients with HF with reduced ejection fraction, who were hospitalised for acute decompensated HF [38]. Also, no differences in the rates of worsening renal function, symptomatic hypotension, and hyperkalemia were observed [38]. Thus, it can be concluded that ARNI might present a valuable therapy option for patients with diabetes and HF.

## ADA/EASD consensus statement 2018: new treatment algorithms for T2DM

On 4 October 2018, new treatment algorithms for T2DM were published based on knowledge gained from CVOTs [39]. In contrast to previous suggestions, the new treatment algorithm recommends a highly patient centred, individualised approach of treatment instead of pushing towards standardised treatment goals. As before, guidelines recommend metformin and lifestyle changes as primary treatment option. Major changes were introduced for second- and third-line therapy: before choosing a second-line therapy, practitioners are encouraged to differentiate between present comorbidities and escalate the therapy accordingly and individually. In case of established CVD, if ASCVD predominates, GLP-1 RA with proven CV benefit or SGLT-2i with proven CV benefit (if eGFR adequate) are recommended. If HF or chronic kidney disease (CKD) predominate, SGLT-2i with evidence of reducing HF and/or CKD progression in CVOTs (if eGFR is adequate) are recommended [39]. In cases without ASCVD, HF or CKD, various choices are offered, depending on the individual patient or setting: if there is a compelling need to minimise hypoglycaemia, if weight gain needs minimising or if costs are a major issue. For injectable therapies, step-wise therapy escalation is recommended, again considering GLP-1 RA options before insulin [39].

## Key topics discussed during the 4th CVOT Summit

For the treatment of diabetes, CVOTs only evaluate effects of selected glucose lowering agents for T2DM, yet not combinatory approaches. Hence, the question of efficacy of these agents (i.e. SGLT-2 inhibitors) in adjunct therapy of T1DM was addressed. Last but not least, parallel to this year’s CVOT Summit, a FDA advisory board re-evaluated the benefit and perpetuation of CVOTs. Likewise, this was debated at the 4th CVOT Summit.

### Adjunct therapy in T1DM

As in T2DM, T1DM is associated with a considerably increased risk of CV events which were shown to occur at a younger age than in non-diabetic individuals [40]. Variations in glucose level and hyperglycaemia in children with T1DM have been associated with persistent cognitive dysfunction [41, 42] and both, hyper- and hypoglycaemia were linked to various adverse CV events [43, 44], although the relationship of severe hypoglycaemia in T2DM seems to be bi-directional [45]. However, as patients with T1DM are mainly treated with insulin, CV safety of new glucose lowering agents was only investigated in the context of T2DM. As of now, a variety of studies has started to investigate the use of glucose lowering medication typically used in T2DM, like metformin, pramlintide, GLP-1 RA, SGLT-2i and dual SGLT-1 and -2i as adjunctive therapy for T1DM, particularly in patients who have inadequate insulin control and/or are overweight [46].

When looking at GLP-1 RA (liraglutide and exenatide) as adjunct therapy in T1DM, one is confronted with significant inter-study variability regarding reduction of HbA1c, postprandial plasma glucose and insulin doses (summarised in [46]). In the ADJUNCT ONE trial [47], evaluating the use of liraglutide as adjunctive therapy in T1DM, inconsistent results regarding HbA1c reduction and reduction of daily insulin dose were obtained across three liraglutide doses compared to placebo. Adverse events included increased rates of symptomatic hypoglycaemia and an increase in hyperglycaemia with ketosis [47].

Various studies investigated the efficacy of SGLT-2i (empagliflozin [48, 49], dapagliflozin [50, 51], canagliflozin [52] and the dual SGLT-1 and -2i sotagliflozin [53, 54]) in the treatment of T1DM. All studies reported a significant decrease in HbA1c [48–54] and some also reductions in body weight [49, 51–53] and daily insulin dose [49]. Adverse events included an increase in genital infections [51, 52] and diabetic ketoacidosis (DKA) [52]. Strategies for the prevention of DKA need to be further established and defined. The strong educational need of health care professionals and diabetes teams was highlighted.

It can be summarised that, although no direct comparison of T1DM and T2DM can be made, agents demonstrating CV safety in CVOTs may also exert beneficial effects when provided as adjunct therapy in T1DM. However, more and larger studies are needed to evaluate if CV safety or benefit demonstrated for those agents in T2DM, next to reductions in HbA1c, bodyweight and insulin dose, also hold true in T1DM.

### Diabetes comorbidities: “Is the future of the treatment of diabetes with CVD in the hands of general practitioners, diabetologists, cardiologists, nephrologists?”

During the 4th CVOT Summit, the question of whose responsibility the treatment of diabetes with CVD should be in future arose—general practitioners (GPs), diabetologists, cardiologists or nephrologists. All disciplines are tightly interwoven in the field of diabetes, also reflected in the spectrum of available treatment options. Looking at patient numbers only, GPs and diabetologists might treat the majority of diabetes patients. However, CVOTs have provided further knowledge on CV and renal comorbidities, making integration of cardiologists and nephrologists in treatment of diabetes indispensable and/or promote further training of diabetologists in cardiovascular disease and of cardiologists in diabetes. Thus, CVOTs promoted the exchange of knowledge and tightened the close network between disciplines, also reflected in the new ADA/EASD consensus statement and future guidelines.

### CVOTs in diabetes: how should we continue?

On the 10 year anniversary of the FDA “Guidance for Industry” [11] in 2018, a FDA advisory board re-evaluated the benefit and perpetuation of CVOTs, parallel to the 4th CVOT Summit in October 2018. Among the issues addressed by the FDA advisory board were: (1) the impact of the recommendations in the 2008 “Guidance for Industry” on the assessment of CV risk for drugs indicated to improve glycaemic control in patients with T2DM; (2) the transferability of CV safety findings from members of a drug class to the entire class of drugs, and (3) whether an inacceptable increase in CV risk needs to be excluded for all new drugs to improve glycaemic control in patients with T2DM, regardless of the presence or absence of a signal for CV risk in the development program [10]. The FDA panel voted for continuation, yet improvement of CVOTs [55].

Questions of similar manner were discussed at the 4th CVOT Summit. On the one hand, positive aspects of CVOTs were reflected by, for example, the detection of unexpected benefits as observed in EMPA-REG OUTCOME [20], CANVAS [21], DECLARE-TIMI 58 [28], LEADER [17], SUSTAIN-6 [18] and Harmony Outcomes [27]. These benefits often are not restricted to CV endpoints; e.g. the CANVAS trial revealed a positive effect of canagliflozin on renal outcomes [21]. These safety and benefit analyses led to the refinement of treatment algorithms as stated in the 2018 ADA/EASD Consensus Statement [39] and the integration of new drugs as “preferred” or “safe” second- or third-line therapy into new guidelines [39, 56]. On the other hand, limitations of current CVOTs, such as the lack of generalisability (i.e. participants often are at high risk for a CV event or death, thus not representative for a larger population), relatively short time-lines for assessing potential harms or benefits and the placebo-controlled design of CVOTs [12] were addressed. Room for improvement of cost-effectiveness and cost-sharing options as well as modification of end points and analyses were also discussed [12]. In summary, concomitant with the FDA panel vote, it was concluded that continuation but modification of CVOTs is highly beneficial as they provide safety aspects relevant to all T2DM patients and create a broad body of evidence to base new guidelines and therapies on.

## Conclusion

The 4th CVOT Summit of the D&CVD EASD Study Group discussed key results of recently completed and published CVOTs in T2DM (CARMELINA, Harmony Outcomes, and DECLARE-TIMI 58) and CV safety studies of PCSK-9 inhibition (ODYSSEY OUTCOMES) in an interactive, multi-disciplinary format. The summit considered both potentials and limitations of current CVOT designs as well as the implementation of CVOTs in the newly published guidelines by the ADA/EASD consensus statement. Learnings for adjunct therapy of T1DM and continuation and modification of CVOT trials were discussed. The D&CVD EASD Study Group will continue its activity. In-depth discussions and presentations of upcoming CVOTs like REWIND, PIONEER-6, VERTIS CV Study or CREDENCE, will be resumed at the 5th CVOT Summit, which will be held from 24–25 October 2019 in Munich (http://www.cvot.org).

## Abbreviations

3P-MACE: 3-point major adverse cardiovascular event
4P-MACE: 4-point MACE
ADA: American Diabetes Association
ACCORD: Action to Control Cardiovascular Risk in Diabetes
ARNI: angiotensin-receptor–neprilysin-inhibitors
ASCVD: atherosclerotic cardiovascular disease
CANVAS: Canagliflozin Cardiovascular Assessment Study
CARMELINA: Cardiovascular and Renal Microvascular Outcome Study With Linagliptin in Patients With Type 2 Diabetes Mellitus
CI: confidence interval
CKD: chronic kidney disease
CV: cardiovascular
CVD: cardiovascular disease
CVOT: cardiovascular outcome trial
DECLARE: Dapagliflozin and Cardiovascular Outcomes in Type 2 Diabetes
DEPICT-1: efficacy and safety of dapagliflozin in patients with inadequately controlled type 1 diabetes
DEVOTE: Trial Comparing Cardiovascular Safety of Insulin Degludec versus Insulin Glargine in Patients with Type 2 Diabetes at High Risk of Cardiovascular Events
DPP-4i: dipeptidyl-peptidase-4 inhibitors
eGFR: estimated glomerular filtration rate
ELIXA: Evaluation of Lixisenatide in Acute Coronary Syndrome
EASD: European Association for the Study of Diabetes
EMA: European Medicines Agency
EXAMINE: Examination of Cardiovascular Outcomes with Alogliptin versus Standard of Care
EXSCEL: Exenatide Study of Cardiovascular Event Lowering
FDA: Food and Drug Administration
GLP-1 RA: glucagon like peptide-1 receptor agonist
Harmony Outcomes: albiglutide and cardiovascular outcomes in patients with type 2 diabetes and cardiovascular disease
HbA1c: glycated haemoglobin A1c
HDL: high-density lipoprotein
HF: heart failure
HR: hazard ratio
LEADER: Liraglutide Effect and Action in Diabetes: Evaluation of Cardiovascular Outcome Results
LDL: low-density lipoprotein
MI: myocardial infarction
NT-proBNP: brain natriuretic peptide
ORIGIN: Outcome Reduction With Initial Glargine Intervention
PARADIGM-HF: Prospective Comparison of ARNI [Angiotensin Receptor–Neprilysin Inhibitor] with ACEI [Angiotensin-Converting-Enzyme Inhibitor] to Determine Impact on Global Mortality and Morbidity in Heart Failure Trial
PCSK-9i: proprotein convertase subtilisin/kexin type-9 inhibitor
REMOVAL: Cardiovascular and metabolic effects of metformin in patients with type 1 diabetes
SAVOR: Saxagliptin Assessment of Vascular Outcomes Recorded in Patients with Diabetes Mellitus
SGLT-2i: sodium/glucose co-transporter-2 inhibitors
SUSTAIN-6: Trial to Evaluate Cardiovascular and Other Long-term Outcomes with Semaglutide in Subjects with Type 2 Diabetes
T1DM: type 1 diabetes mellitus
T2DM: type 2 diabetes mellitus
TECOS: Trial Evaluating Cardiovascular Outcomes with Sitagliptin
UGDP: University Group Diabetes Program
US: United States
